# Artificial Neural Network with a Cross-Validation Technique to Predict the Material Design of Eco-Friendly Engineered Geopolymer Composites

**DOI:** 10.3390/ma15103443

**Published:** 2022-05-10

**Authors:** Yaswanth Kuppusamy, Revathy Jayaseelan, Gajalakshmi Pandulu, Veerappan Sathish Kumar, Gunasekaran Murali, Saurav Dixit, Nikolai Ivanovich Vatin

**Affiliations:** 1 Department of Civil Engineering, B.S. Abdur Rahman Crescent Institute of Science & Technology, Chennai 600048, Tamil Nadu, India; yaswanthkkphd@gmail.com (Y.K.); gajalakshmi@crescent.education (G.P.); 2 Faculty of Civil Engineering, Architecture and Geodesy, University of Split, 21000 Split, Croatia; 3 Peter the Great St. Petersburg Polytechnic University, 195251 St. Petersburg, Russia; murali_22984@yahoo.com (G.M.); sauravarambol@gmail.com (S.D.); vatin@mail.ru (N.I.V.); 4 Division of Research & Innovation, Uttaranchal University, Dehradun 248007, Uttarakhand, India

**Keywords:** artificial neural networks, cross validation, engineered geopolymer composites, mix design

## Abstract

A material-tailored special concrete composite that uses a synthetic fiber to make the concrete ductile and imposes strain-hardening characteristics with eco-friendly ingredients is known as an “engineered geopolymer composite (EGC)”. Mix design of special concrete is always tedious, particularly without standards. Researchers used several artificial intelligence tools to analyze and design the special concrete. This paper attempts to design the material EGC through an artificial neural network with a cross-validation technique to achieve the desired compressive and tensile strength. A database was formulated with seven mix-design influencing factors collected from the literature. The five best artificial neural network (ANN) models were trained and analyzed. A gradient descent momentum and adaptive learning rate backpropagation (GDX)–based ANN was developed to cross-validate those five best models. Upon regression analysis, ANN [2:16:16:7] model performed best, with 74% accuracy, whereas ANN [2:16:25:7] performed best in cross-validation, with 80% accuracy. The best individual outputs were “tacked-together” from the best five ANN models and were also analyzed, achieving accuracy up to 88%. It is suggested that when these seven mix-design influencing factors are involved, then ANN [2:16:25:7] can be used to predict the mix which can be cross-verified with GDX-ANN [7:14:2] to ensure accuracy and, due to the few mix trials required, help design the SHGC with lower costs, less time, and fewer materials.

## 1. Introduction

Engineered geopolymer composites (EGC) are a new class of ultrahigh ductile fiber–reinforced concrete composites with 100% eco-friendly binding materials [1]. Geopolymer concrete uses industrial by-products such as fly ash (FA), ground granulated blast furnace slag (GGBS), and metakaolin (MK) as binding materials that are activated by alkali activators [2]. Combinations of hydroxides and silicates of sodium, potassium, or calcium, in various proportions, are generally used to activate aluminosilicate-based binders. These geopolymeric binders are reinforced with special synthetic fibers, such as polyvinyl alcohol (PVA) fibers or polyethylene (PE) fibers, that constitute EGC. The EGC exhibit peculiar strain-hardening characteristics, thus making the concrete ductile rather than brittle. The ductility of EGC is nearly 600 times that of normal concrete [3]. These material ingredients of the composites are tailored in such a way as to obtain multiple micro-cracks upon loading, making the concrete flexible in nature. EGC is popularly known as bendable concrete, flexible concrete, or strain-hardening geopolymer composite (SHGC). Several studies are being carried out in the field of EGC all over the world, and these include advanced experiments. The state-of-the-art research on EGC includes studies on the effects of different kinds of binders; on the blending proportions, i.e., the binary binders in EGC [4]; on the effects of nano materials on the interfacial properties of EGC [5]; and a few durability studies on the effects of nano materials [6]. Such advanced experiments require substantial costs and time.

Designing concrete composites to achieve the desired compressive strength is always tedious. The mix-design procedure of conventional concrete has been standardized such that the concrete can be designed to achieve the target compressive strength [7]. Research into concrete technology has been extensively developed, resulting in the development of several special concrete-like high-performance concretes [8,9], fiber-reinforced concrete [10,11,12], self-healing concrete [13], lightweight concrete [14,15], etc. During the development stages of such special types of concrete, it is always required for the researchers to perform several trial mixes in a feasibility study and to achieve the desired strength, which may consume a huge amount of materials and time. Since EGC is in the developmental stage, the scenario for trial mixes of the material still exists.

The artificial neural network (ANN) is one of the artificial intelligence tools widely applied in almost all kinds of research worldwide [16,17,18]. The ANN is based on the principle of millions of interconnected human biological neurons that respond based on signals from the brain. This technique is applied in ANN to predict responses based on several inputs by establishing a non-linear relationship. In civil engineering research, ANN has been successfully applied to anticipate the strength criteria of hardened concrete [19] and the workability properties of fresh concrete [20]. In addition to these studies, several important and advanced parametric studies, such as those estimating the bond strength of structural concrete [21,22], the spalling [23] damage assessment of concrete [24,25], analyzing sections of deep beams [26], estimating the fracture parameters of geopolymer composites [27], and accessing the properties of FRP columns [28] have been successfully conducted. Additionally, durability studies on various factors such as corrosion inhibition [29], chloride penetration [30,31] and other aspects have also been successfully undertaken. One of the rare phenomena in applying ANN to concrete technology is the optimization of the mix-design process of special types of concrete [32,33,34]. A few researchers have successfully applied the ANN technique to predict and optimize the mix design of conventional and special concrete; however, standardizing the procedure and conformity of the prediction abilities of those ANN models is still tedious.

## 2. Research Significance

In the early stages of the developmental phase of advanced research on special concrete and in order to study the material’s behavior in relation to various properties such as high strength and durability, it is essential to preliminarily understand the material’s basic behavior. The paramount concern in designing any kind of concrete composite is the compressive strength. For the conventional type of concrete, standard mix-design procedures are available by which, with the available material properties such as specific gravity and gradation, among others, the concrete composite can be designed to achieve the desired compressive strength [7]. For special concrete composites, the mix design can be tedious to standardize. For instance, to design self-compacting concrete, the dosage and the type of super-plasticizer primarily depend on the binder, where several supplementary cementitious materials may also be used along with the cement. In such cases, it is impossible to standardize all the types of binding materials. Thus, trial mixes are the only true methods of possibly developing the composites. The problems associated with the trial mixes are the massive consumption of materials and time for the design, which are not economical and may delay the progress of advanced research.

To overcome this, several advanced techniques are being adopted in concrete technology to standardize the procedure of design [35]. Among them, artificial intelligence is the most prominent. Since the early 2000s, soft computing techniques (SCT) have been applied in civil engineering, particularly in predicting the strength parameters of concrete composites [17,36]. Various mix-design factors are identified, numbered, and specified as inputs to anticipate the compressive strength of the composites. Specifying those inputs and outputs should determine those mix-design factors, technically known as the “design mix of concrete”. The application of SCTs in concrete mix design is a rare phenomenon and the accuracy of these predictions has not been addressed [34].

EGC is one of the types of special concrete for which standards have not been prescribed, and since it is in the initial phases of development, several advanced studies are being carried out worldwide [37,38]. The available literature on the development of EGC is quite limited such that performing future studies on EGC requires more trials since assumptions cannot be made for any aspects. Using synthetic fibers in EGC makes it quite uneconomical [39]. Thus, with the help of advanced computing techniques, a mix-design procedure is a prerequisite to minimizing the materials and time consumption in the trial mixes of EGC material development. This paper attempts to develop a mix-design procedure using the ANN technique. In this research work, Levenberg Marquardt (LM)–based ANN is employed to predict the mix-design factors. The GDX (gradient descent adaptive LR)–based ANN is adopted to analyze or cross-validate the mix-influencing factors. With the various types of ANN models, such a type of cross-validation enhances the accuracy of prediction and validation. Thus, it helps to minimize the number of trial experiments to achieve the desired strength of special concrete composites. The cost and time required for the experiments can also be reduced.

## 3. Methodology

**Figure 1:**
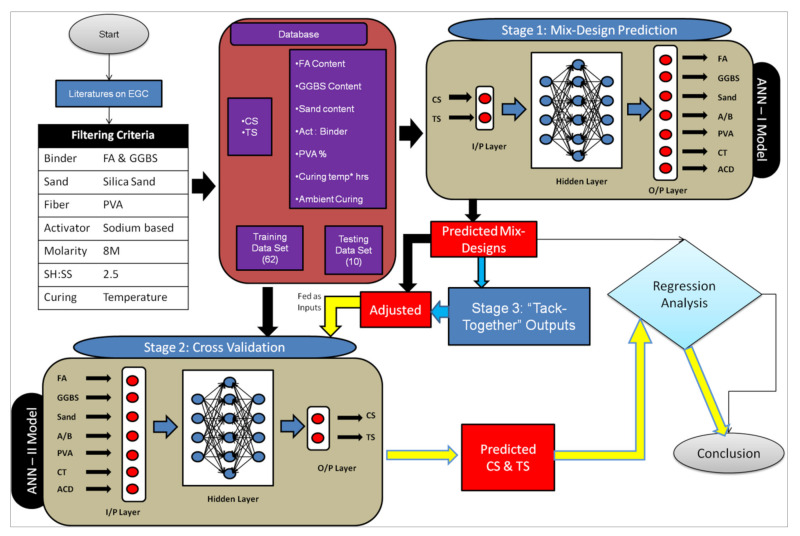
Methodology of research.

Figure 1 shows the detailed methodology that was performed in this research study. This paper adopts the ANN to predict the mix-design influencing parameters in developing SHGC for the desired compressive strength and tensile strength. In other words, this is a detailed study on the mix-design process of developing SHGC using an ANN when a standard mix-design procedure is not available. The methodology comprises three stages: prediction, cross-validation and tack-together’ output for a preliminary database collection.

As a preliminary stage, a database required for training, testing, and cross-validating the ANN models was prepared, which was collected from the EGC papers, with a few ‘literature filtering’ criteria. It is obvious that the presence of surplus mix-design factors or materials or the involvement of the addition or replacement of mix-design ingredients (apart from those seven selected mix factors) may certainly influence the compressive strength and tensile strength. Thus, it was required to select the literature in such a way that no other influencing factors were involved, for which the filtering criteria were adopted. For instance, the fine aggregate selected here was silica sand, but some researchers might have used river sand in EGC, along with GGBS/fly ash, so such studies were filtered in the data collection process as the performance of silica sand and river sand may vary upon compressive strength. In the first stage, as per the computing techniques, parameters obtained once the concrete has been prepared in the fresh or hardened state can be given as inputs to predict the mix factors [34]. Thus, parameters such as slump values, strength values, or Young’s modulus can be given as inputs. As per the literature available and the database collected, two strength parameters, compressive strength and tensile strength, were given as inputs, whereas the seven mix-design influencing factors—fly ash content, GGBS content, sand content, activator/binder ratio, PVA V_f_ (%), curing temperature x hours, and ambient curing days—were given as outputs. Several ANN models were created and analyzed to predict the seven mix-design factors, with only compressive strength and tensile strength values as inputs. Predicting seven outputs with only two inputs is a tedious task as per ANN [40]. Thus, the trial-and-error method was adopted to train and test the various ANN models with variations in a number of hidden layers and hidden neurons in it, of which the five best ANN models were chosen (named ANN-I models). The predicting ability of those ANN-I models was ensured through regression analysis with coefficient of determination (R^2^) as performed in the previous literature [41,42].

In the second stage, to test and validate the created ANN-I models, another type of ANN model was developed, the ANN-II model, which predicts the compressive and tensile strength with those seven mix-design influencing factors as inputs. Now, to validate the ANN-I models, the mix-design outputs obtained were adjusted and given as inputs to the trained ANN-II model to predict the compressive strength rearwards, denoted as “cross-validation” as mentioned by Jena et al. 2020 [43]. The term ‘adjustments’ indicates the corrections that can be made in the predicted mix-design values obtained from the ANN-I models. Certain outputs may go beyond the allowable range during the prediction of mix-design values from ANN-I models. For example, it is redundant if the output activator/binder ratio is beyond 1.3. These values were then adjusted to the nominal value of 0.65. It is to be noted that such adjustments were made only to the activator/binder ratio and PVA fiber (%) (Taken as 2, if >2) and the remaining outputs were fed unchanged. This predicted compressive and tensile strength would be analyzed with the experimental database obtained from literature to test the predictive ability of the ANN-I models.

The third stage involves a method known as “tack-together output”. Despite considering all the seven outputs (mix-design influencing factors) from the same ANN-I models, each output with the maximum R^2^ value was isolated and tacked together. For example, the fly ash content’s first output may show the maximum accuracy from any one of the five ANN-I models, which is isolated. Similarly, other outputs were also isolated and these isolated outputs were tacked together to form a combined mix design. These combined mix-design outputs were again validated in the second stage of cross-validation with the ANN-II model and the results were analyzed.

## 4. Database

The literature based on the material studies of engineered geopolymer composites (EGC) was collected. In total, 14 studies [38,39,44,45,46,47,48,49,50,51,52,53,54,55] were selected as per the criteria mentioned in Table 1 and the database was framed. A total of 72 datasets were collected from the carefully selected 14 studies with the aforesaid mix-influencing factors. The research on material EGC started initially as a feasibility study by Onho et al. in 2014 [1]. Researchers worldwide performed various material and behavioral studies on EGC on the aspect of developing strain-hardening concrete composites and the importance of sustainability. Hence, the data available concerning such novel material are comparatively limited. Since only a limited number of inputs are chosen as mix-design factors, studies involving only those selected mix factors can be considered, which further limits the datasets. It is also to be noted that, the studies involving surplus mix design factors were not considered for data collection, i.e., for instance, if a study uses metakaolin or silica fume as one of the binders (which are other than the selected binders of fly ash and ggbs), then the study was not considered for data collection. This was certainly to improve the accuracy of prediction. Furthermore, studies like A Yaman et al. 2017 [34] proved that adequate mix-design accuracy can be obtained for developing ANN models. The researcher used 69 datasets for both training and validation in predicting the mix-design and showed an accuracy level of 80%. The mix-design influencing factors such as binder contents, fiber dosage, curing conditions, etc., were initially selected for which the prediction needs to be made and is described in Table 1.

The mix-design influencing factors selected were based on the efficiency of those materials and the availability of experimental data. Several kinds of study on EGC have preferred metakaolin (MK) as binding material; however, the availability of experimental data was comparatively lower, though the efficiency of using MK is similar to FA and GGBS. Similarly, strain-hardening criteria can be achieved by polyethylene and polyolefin fibers, but PVA fibers are most efficient as per the literature. Several variations on geopolymer activators, such as the molarity of sodium hydroxide and the ratio of sodium hydroxide to silicate, could be identified in the literature. Only the efficient combinations were selected, as specified in Table 1. The curing conditions chosen were high-temperature curing followed by ambient curing until the test.

In stage 1, to predict the mix-design for SHGC, the inputs were compressive strength (CS) and tensile strength (TS) and the outputs were seven mix-design factors. The specifications of the collected dataset, i.e., its minimum–maximum range, and standard deviation for both training and testing is presented in Table 2. This could help to identify the quality of data collection. For the second stage of cross-validation, the inputs and outputs become reversed. Among the 72 datasets, 62 datasets were used for training the models and a subset of 10 datasets was used for testing the predictive models.

## 5. Development of ANN Models

### 5.1. Architecture of ANN Models

**Figure 2:**
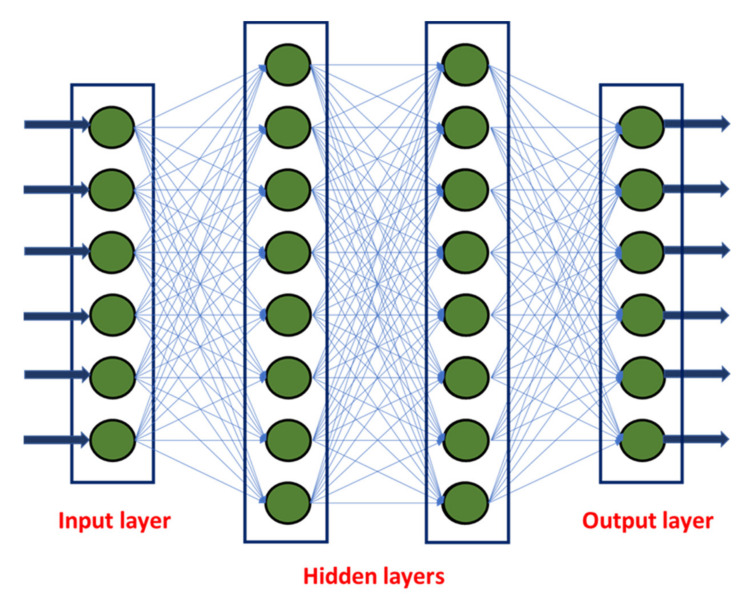
ANN Architecture.

As described earlier, ANN works on the principle of human/animal biological neurons, i.e., several million neurons are interconnected between the brain and other parts of the body, and based on the signal transmission, the brain recognizes and instructs. Similarly, ANN consists of three layers: an input layer, hidden layer(s), and an output layer. The data for input and output layers were collected as described in Section 4. Now, the development of best models lies in formulating the number of hidden layers and number of hidden neurons in each layer; selecting the right type and function of ANN; and developing criteria such as epochs and max-fail and others, which were mentioned in Section 5.2. Figure 2 shows the basic architecture of ANN models. Based on the number of inputs, outputs, and hidden layers, an empirical relation between inputs and outputs is established based on the weights associated with the links, which is represented in Equation (1)

O_1_ = f [(W_1_∗H_1_) + (W_2_∗H_2_)…(W_n_∗H_n_). I_1_] +……+ f [(W_N2_∗H_N2_) + (W_N2_∗H_N2_)…(W_Nn_∗H_Nn_). I_n_] (1)

Here, O represents output; W represents weights; I represents inputs; H represents hidden neurons.

Now, to calculate an output, which is related to hidden neurons and inputs, the weights associated with the empirical relation has to be assigned with several trials until good accuracy in the equation is obtained, which is called training. MATLAB software was used in this study to establish best equations (i.e., best ANN models) with several trials and iterations. With those established equations, outputs can be further predicted.

### 5.2. Stage 1: Prediction of Mix-Design Influencing Factors

After the development of the database, it was essential to select the type of ANN network and its required criteria for training the models. Undoubtedly, the feed-forward backpropagation type of ANN network was chosen based on the literature’s acknowledgments [17,36]. Based on the trial-and-error method, several functions of neural networks were tried and it was found that LM-based ANN worked best in predicting the mix design. As the LM-ANN worked well in predicting the outputs when the number of inputs was comparatively smaller [56]. To obtain the best ANN models in predicting the mix-design, variations in the number of hidden layers and the number of hidden neurons were considered and trained accordingly.

During the training stages, 70% of the dataset was used for training, 15% for self-validation, and 15% for self-testing as per the “*nntool”* algorithm. It is to be noted that the training criteria, such as the number of epochs, max_fail, and other factors, vary for different ANN models. The learning and transfer functions used were “LEARNGDM” and “LOGSIG”, respectively. Starting with a single hidden layer, hidden neuron ranges from 0 to 20 were tried. ANN [2:16:7] (indicated as 2 inputs; 1 hidden layer with 16 neurons and 7 outputs) resulted in nominal values of material conditions and curing regimes. It was identified that the higher the number of hidden neurons, the higher its prediction accuracy. However, numbers greater than 16 (>16) was not considered since the training process failed upon regression.

**Figure 3:**
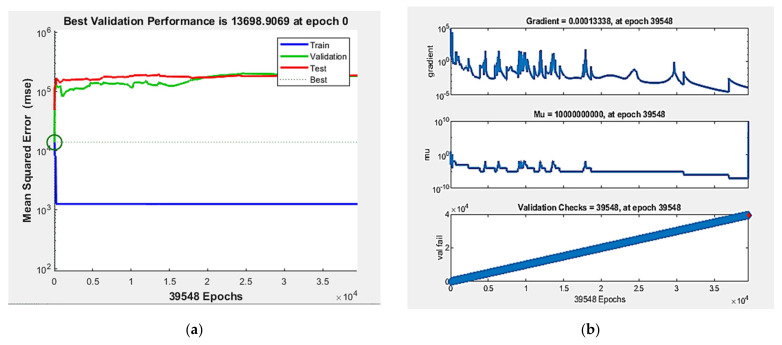

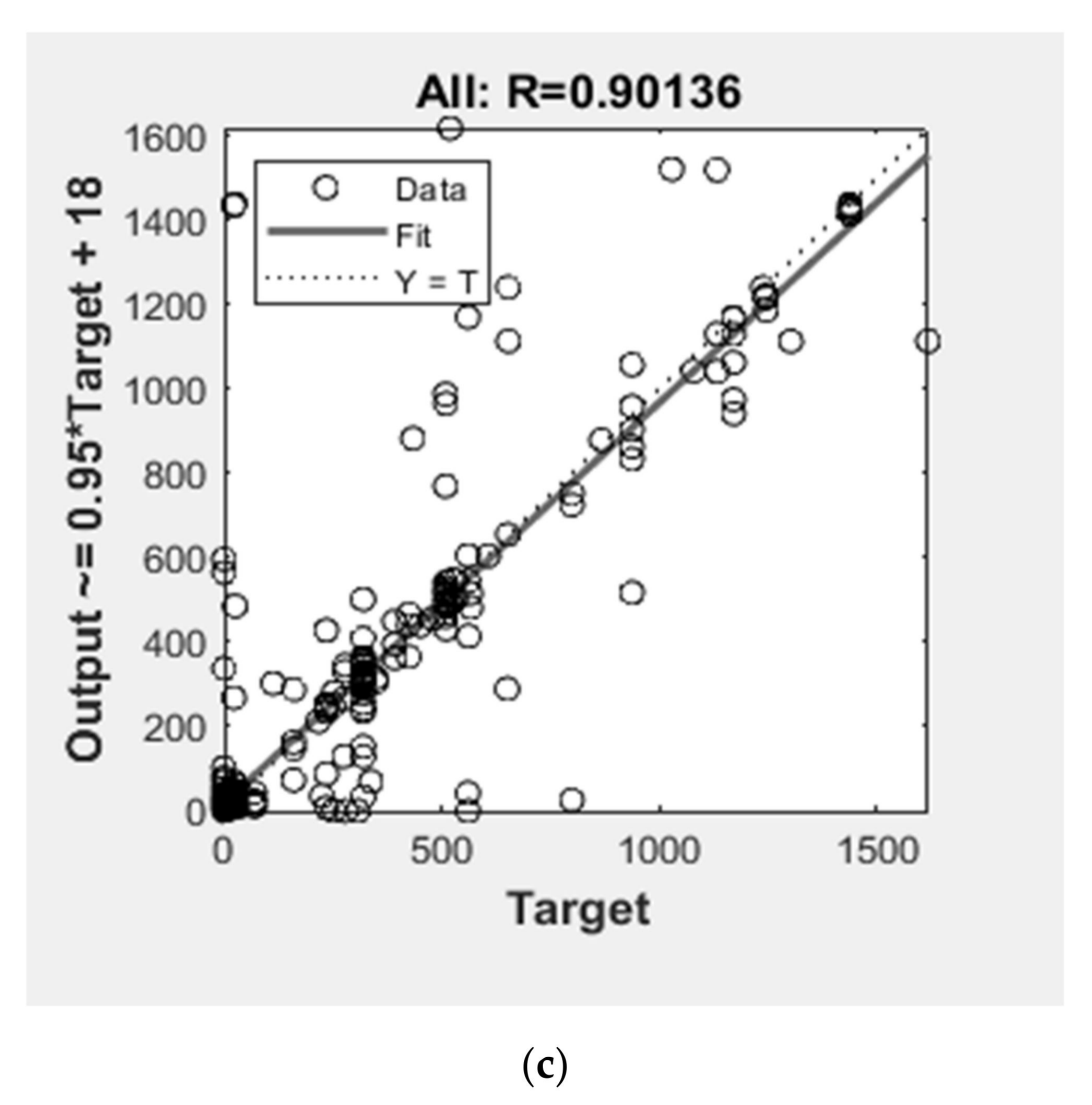
Training performances of ANN [2:16:16:7] model, (**a**) nest validation performance; (**b**) training state; (**c**) regression.

In the next phase, two layers of hidden neurons were used. Under different combinations of hidden neurons, it was found that the first hidden layer with 16 neurons was more efficient than the other combinations. Keeping 16 neurons as constant for the first hidden layer, various numbers of hidden neurons upon hidden layers were tried and self-tested and it was found that the ANN [2:16:16:7] and ANN [2:16:25:7] models provided nominal results. Furthermore, various hidden neurons with three hidden layers were tried and found the best possible outcomes as ANN [2:16:16:8:7], ANN [2:16:16:25:7] and ANN [2:16:32:16:7] models. Table 3 summarizes the correlation coefficient (R) obtained during the training stages with the best possible ANN models. Among those ANN models, the best ANN model developed was identified to be the ANN [2:16:16:7] model with the best analysis upon a correlation coefficient during the training stage. Figure 3 depicts the best validation performance, training state, and regression obtained for the ANN [2:16:16:7] model.

### 5.3. Stage 2: Prediction of Compressive and Tensile Strength for the Cross-Validation of ANN-I Models

**Figure 4:**
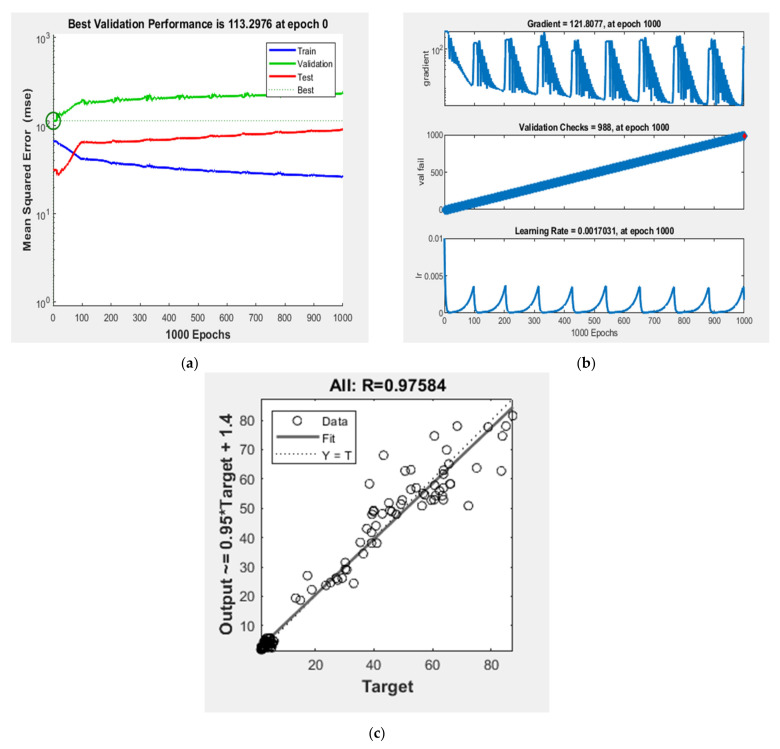
Training performances of ANN [7:14:2] model, (**a**) nest validation performance; (**b**) training state; (**c**) regression.

In this stage, the same 72 datasets were employed in the development of the ANN-II model to predict the compressive and tensile strengths. During the prediction of compressive strength with several mix-design factors, i.e., few outputs with several inputs, GDX based ANN performed best, as per the literature [57]. Hence, initially, one hidden layer with several hidden neurons was trained and tested for its predictive ability based on the trial-and-error method. It is to be noted that the databases used for training and testing, respectively, for ANN-I models were used in ANN-II also. It was found that ANN [7:14:2] performed best with predictive ability, up to 94%. Performances during the training process of the ANN [7:14:2] model are shown in Figure 4. For cross-validating, the mix designs obtained from the ANN-I models were adjusted and fed as inputs to the ANN-II model and the compressive strength and tensile strength were predicted. Thus, the predicted compressive and tensile strength from the ANN-II model was analyzed with the experimental literature database for validation. It thus helped to verify the predicting ability of the ANN-I models.

## 6. Results and Discussion

ANN-I models were developed to predict the numerical mix-design factors that help develop the material SHGC for its desired compressive and tensile strength. The five best models were selected from several trained models. The analysis on the predicting ability of ANN models to mix-design factors does not primarily lie in regression analysis, unlike compressive strength. For instance, when predicting sensitive parameters such as compressive strength, variance beyond ±5% is not acceptable [7], whereas predicting outputs such as binder content can be accepted within the range of ±20% [34]. Thus, the regression analyses were done with the perfect line analysis of range ± 20% variance. The combined analysis is the most important thing to consider in analyzing the predictive ability of ANN models on mix-design. Mix design is the combination of several factors/parameters towards a target. Change in one of the parameters correspondingly when there is a change in other parameters(s) may result in the same target. In other words, for a single target, there may be several combinations of inputs feasible. The ratio between the binders and aggregates may be constant, but the content/value varies. Due to this fact, regression analysis of the ANN models alone is inadequate to examine the ability of prediction, for which the cross-validation was performed. However, the regression analyses were performed initially, followed by cross-validation, for preliminary logical checks.

### 6.1. Regression Analyses of Mix-Design Prediction of ANN-I Models

The regression analysis with the coefficient of determination (R^2^) was performed for the predictions of mix-design obtained from the five best ANN-I models. The prediction results and the experimental literature database are summarized through Table 4a–g with the coefficient of determination values. The results have been summarized as individual outputs of five ANN-I models. As mentioned earlier, the prediction of mix-design influencing factors with the variance of ±20% is quite acceptable, i.e., a coefficient of determination greater than 80% is considered to be the best result. Thus, perfect line analysis showing the variance of ±20% lines has been included in regression plots. However, a smaller coefficient of determination does not mean that the prediction is inaccurate or has lower predictive ability, for which the cross-validation was performed.

**Figure 5:**
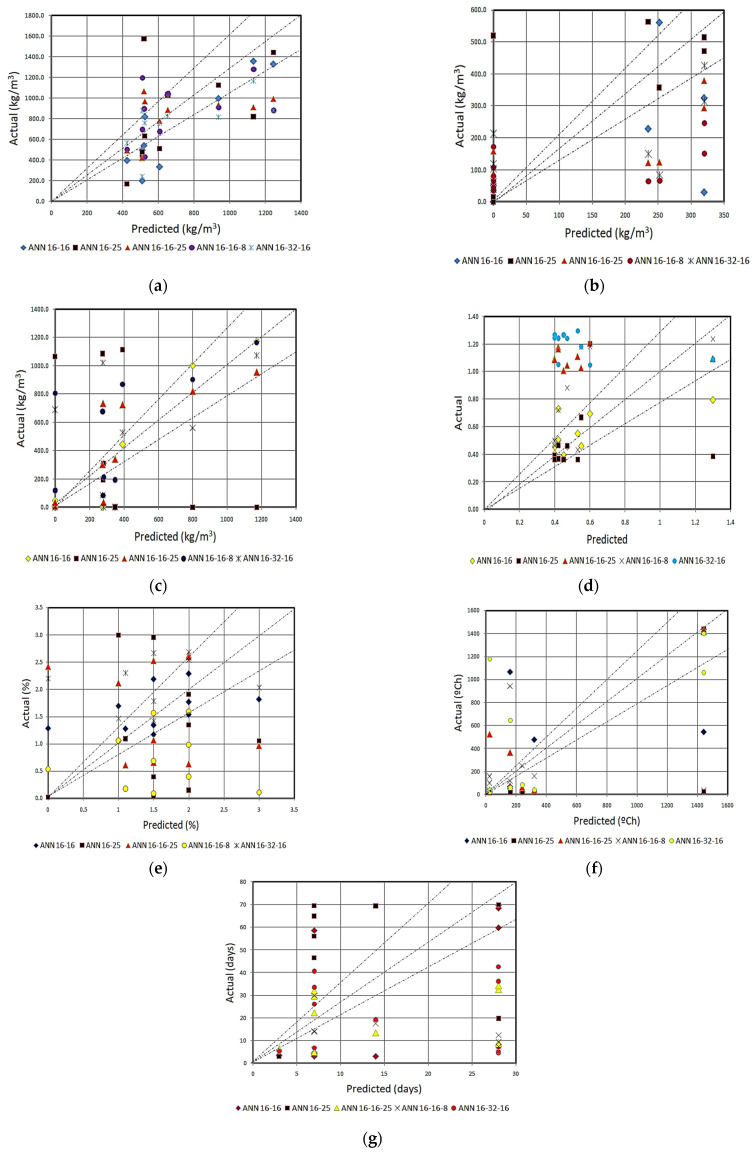
(**a**) Fly ash prediction; (**b**) GGBS prediction; (**c**) sand prediction; (**d**) activator/binder ratio; (**e**) PVA fiber (%) prediction; (**f**) curing temperature x hours prediction; (**g**) ambient curing days prediction.

The fly ash content was predicted with the maximum accuracy of 75% with ANN [2:16:16:7] model upon regression analysis with the validation dataset, as shown in Figure 5a. The highest prediction accuracy was obtained with the sand content prediction with the same ANN model with an accuracy of 85%. Similarly, looking upon GGBS prediction with the same ANN model, it was observed to be 48%, which is quite phenomenal. Thus, it is inferred that ANN [2:16:16:7] model performs well in predicting the material contents. The material contents and their proportion form the major part of the mix design and it is suggested to use the model in determining the mix proportion of binding and aggregate materials. As per Figure 5a of the perfect line analysis, it is observed that most of the predictions fall within the ±20% range.

As far as the GGBS content prediction is concerned, 62% accuracy was achieved with ANN [2:16:16:25:7] model. The best prediction achieved was with ANN [2:16:16:7] model concerning the arithmetic error, i.e., with the validation dataset, this ANN model recognized wherever “0” content was exhibited and the arithmetic variance of other datasets were quite acceptable. In predicting the GGBS content, all the five ANN-I models performed well with 50% accuracy in regression, as shown in Figure 5b. Briefly, 85% and 70% accuracy were obtained in predicting the sand content with ANN [2:16:16:7] and ANN [2:16:16:25:7], respectively, as per Figure 5c. The former model is suggested in predicting the sand content since it was the most notable achievement in prediction [21].

The prediction of the mix-design factor for activator/binder ratio shows two accumulations throughout the scatter plots, as shown in Figure 5d. One of the accumulations lies within the 20% boundary, whereas the other group of values was a little far away from the acceptable range. Whereas predicting the PVA fiber percentage shows the distribution of values/results throughout the graph, only a few percent of the results are within the acceptable range, as shown in Figure 5e. This is primarily because the ranges of these inputs were very low and the availability of an experimental literature database is not adequate. It is suggested to use the nominal values for activator/binder ratio and PVA volume fraction of the design of SHGC.

As shown in Figure 4f,g, most predictions lie outside the 20% range. Though the ranges of the output “curing temperature x hours” is large as indicated in Table 2, the values in the dataset were not uniformly distributed. 50% of the datasets lie within 25 °C.h whereas the other datasets were close to 1440 °C.h. The availability of large databases for training would help to avoid such inaccurate predictions. The ANN [2:16:16:25:7] model predicted the output with the accuracy of 86%, as per Table 4f, but it was difficult to recognize whether to go for the type of curing. As per Table 4g, ANN [2:16:16:7] could only predict the curing days to some extent, but the other models could not produce any significant results. The outputs obtained for this particular mix factor “curing temperature x hours” can be viewed in two ways. For output “100”, does it mean 100 °C for 1 h or 25 °C for 4 h? In geopolymer technology, the choice of ambient curing for long curing days or elevated temperature curing with a shorter duration depends entirely on the structural application [51]. Similarly, using the nominal curing temperature and curing type is suggested based on its structural application; however, the ANN models gave the optimized duration for the desired mix.

### 6.2. Regression Analysis of Validation of ANN-II Model

**Figure 6:**
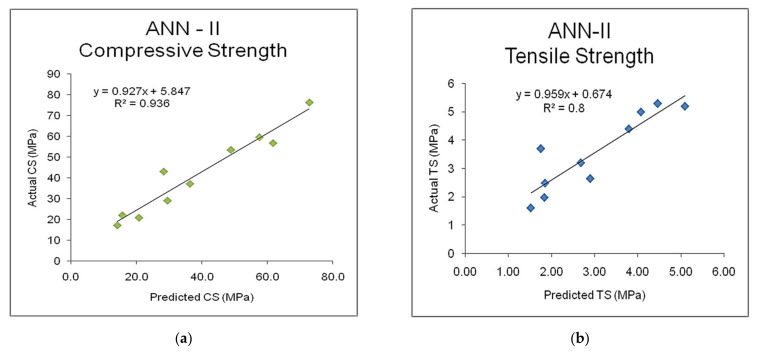
Regression analysis of (**a**) compressive strength (CS) and (**b**) tensile strength (TS) of ANN-II model.

A GDX based ANN model was trained and validated with seven mix-design factors as inputs and compressive and tensile strength as outputs. To test the predictive ability and cross-validating ability of the ANN-II model, the validation dataset was also used, and the predicted results are compared with the experimental literature database. Table 5 shows the comparison between the predicted output and literature experimental database along with the coefficient of determination, and root-mean-squared error (RMSE) upon testing the model. It is observed that overall 94% accuracy was obtained with the ANN-II model in predicting the compressive strength with those seven mix-design factors as inputs. It is adequate to check for the arithmetic error between the prediction and original data for more sensitive parameters like compressive strength. As given in Table 5, on average, a magnitude of four was obtained as RMSE, which is quite acceptable. The regression analyses of the predicted outputs of compressive and tensile strengths and the experimental literature database are illustrated in Figure 6a,b, respectively.

### 6.3. Tack-Together Outputs

Based on the regression analysis of mix-design prediction, each factor is isolated upon maximum coefficient of determination and tacked-together. The predicted outputs ANN [2:16:16:7] model showed the maximum accuracy of 74% in predicting the fly ash content; thus from the output fly ash content, the predicted results of ANN [2:16:16:7] were chosen. The same model’s prediction for the output sand content also was chosen because of its maximum accuracy of 85%. Similarly, the other five mix-design factors were also isolated and tacked together.

Adjusted values were taken for outputs activator/binder ratio and PVA fiber (%). For curing temperature x hours, ANN [2:16:16:25:7], and for ambient curing days, ANN [2:16:16:7] were chosen for its maximum accuracy. However, adjustments to the nominal values can be made wherever required, as described earlier. The tacked-together outputs of mix design are shown in Table 6, which was also cross-validated to test its accuracy.

### 6.4. Cross-Validation of Mix Design and ‘Tacked-Together (TT)’ Outputs

The predicted mix design from ANN-I models (illustrated in Table 4a–g) was given as inputs to the ANN-II model that can anticipate the compressive strength, i.e., the outputs obtained from LM-ANN (ANN-I) models were given as inputs to the GDX-ANN (ANN-II) model and the compressive strength was predicted, which was analyzed with the original experimental data as cross-validation. Apart from this, the combination of mix design obtained from the tacked-together model was also analyzed. The predicted compressive and tensile strength of ANN-II model as cross-validation, along with the literature experimental database upon regression analysis and RMSE are presented in Table 7a,b.

**Figure 7:**
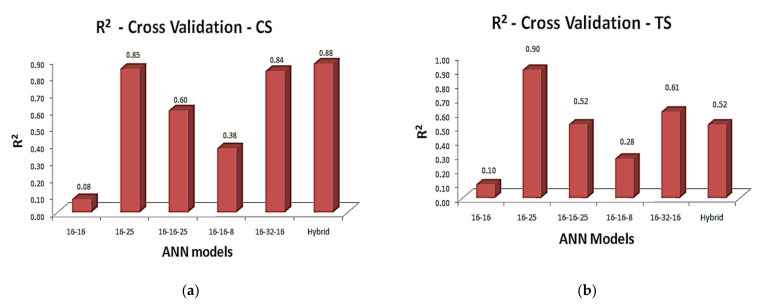
(**a**): Regression analysis of cross-validation of ANN-I and tacked-together models; (**a**) compressive strength, (**b**) tensile strength.

The regression analysis with a coefficient determination of ANN-I models and the hybrid model upon cross-validation for compressive and tensile strength are illustrated in Figure 7a,b, respectively. Some interesting results could be observed upon cross-validation. For ANN [2:16:16:7] model, five outputs out of seven showed greater accuracy in terms of regression analysis, which as a whole mix fails in cross-validation, since as per the GDX-ANN model, the mix can give the compressive strength with only 8% accuracy, which is unreliable. In the same way, the ANN [2:16:25:7] and [2:16:32:16:7] models, which were not up to the mark upon regression analysis, showed accuracy of 85% in cross-validation, with the least RMSE, of magnitudes 7.45 and 8.53 respectively.

As discussed in Section 3, the ANN-I models may fail upon individual regression analysis, but the models may perform best as a combination of the seven mix-design factors. Thus, it can be suggested to use those two models to predict the mix-design of SHGC upon the compressive strength with those mix-factors and curing conditions.

As per Table 8, cross-validation of tacked-together outputs shows the accuracy up to 88% upon compressive strength and 52% upon tensile strength. The interesting interpretation here was five mix-design factors of ANN [2:16:16:7] model outputs (best ANN-I model upon regression analysis) and two mix-design factors of ANN [2:16:25:7] outputs (best ANN-I model upon cross-validation) were tacked together, which also performed best upon cross-validation.

The ANN model [2:16:25:7], which performed well upon compressive strength, was also found to be best upon the tensile strength, with the predictive ability of up to 90% with RMSE 0.27, which is phenomenal, as indicated in Table 7b. For materials like EGC, its application in the field is mainly due to its high tensile strain capacity than conventional concrete. Thus, it is mandatory to achieve its desired tensile strength. The ANN model [2:16:16:7] showed better regression analysis, but upon cross-validation, it showed the accuracy only up to 10%, with huge RMSE, which is not highly recommended.

As far as the tacked-together model upon cross-validation is concerned, the combination of individual best results of ANN-I models as a mix also showed greater accuracy up to the level of 88% on compressive strength but showed accuracy of only 54% on tensile strength. Thus, it is also suggested to use the tacked-together output combinations of different trained ANN models to predict the mix-design of SHGC with proper nominal adjustments when compressive strength is the primary concern. It is also to be noted that the ANN-II model showed accuracy of 94% only, i.e., the coefficient of determination obtained from cross-validation may still have improved accuracy beyond the results obtained.

## 7. Conclusions

The ANN technique was attempted to predict the mix-design of SHGC for its desired compressive and tensile strength including curing conditions. Five LM-based ANN models were developed to predict seven mix-design factors based on two inputs, compressive and tensile strengths. The predicted outputs of these five models were analyzed upon regression using a coefficient of determination. The best resulting outputs of those five best models were summarized and “tack-together mix-design” was framed and also analyzed. A GDX-based ANN model was developed, trained, and tested with 94% accuracy to predict the compressive strength for cross-validation. The outputs obtained from LM-based ANN models were fed as inputs to GDX-based ANN for cross-validation. The regression and cross-validation results were analyzed, from which the following conclusions have been summarized.

- This research study reveals that the basic mix-design parameters required to design the material EGC are feasible with few LM-based ANN models which can be cross-analyzed with GDX-based ANN and if only these seven mix-design influencing factors are involved, then ANN [2:16: 25:7] can be used to predict the mix which can be cross verified with GDX-ANN [7:14:2] for ensuring accuracy.
- The five best ANN models that can predict the mix-design of SHGC were LM-based ANN [2:16:16:7], ANN [2:16:25:7], ANN [2:16:16:25:7], ANN [2:16:16:8:7], and ANN [2:16:32:16:7], and the best model for cross-validation was GDX-based ANN [7:14:2].
- A few models, i.e., ANN [2:16:16:7], performed well on regression analysis, which failed to perform cross-validation. This insists on the importance of cross-validating since to predict the mix-design of composites, the performance of the combination of all the factors is the key concern and not the performance of individual factors/materials.
- Even though the ANN [2:16:25:7] model showed less accuracy upon regression analysis, it performed well on cross-validation with the accuracy of prediction up to 85% and 90% upon compressive and tensile strength.
- In addition to the identification of each best mix-design factor upon regression analysis, isolating and tacking-together also performed best with the accuracy of 88% upon cross-validation.
- Thus it is recommended to use those predictive models for the material design of EGC involving the aforesaid mix factors with fewer trial mixes. This would certainly reduce the cost and time of trial experiments. However, the models cannot be applied directly for EGC involving surplus mix-factors.

### Future Scope

The material EGC is in the developmental stage, so there may be the need for analytical and experimental studies in various aspects of EGC, such as material development, structural behaviors, micro-structural enhancements, etc. The utilization of such ANN models reduces the trial mixes in the methodology and finds its way to develop more accurate predictive models, as larger availability of data certainly strengthens the training stage of predictive models. This will allow researchers to develop more accurate predictive models with various other-computing techniques such as response surface methodology, gene expression programming, and other optimization techniques.

## Figures and Tables

**Table 1:** Mix-design parameters, types of materials/criteria adopted in this research.

Mix-Design Parameters	Type of Materials/Curing Criteria
Binders	Class F Fly Ash (FA) and/or Ground Granulated Blast Furnace Slag (GGBS)
Fine Aggregate	Silica Sand
Fiber	Poly Vinyl Alcohol (PVA) only
Activator	Sodium-Based Activators only (8M-NaOH; NaOH:Na_2_SiO_3_-2.5)
Curing	Temperature Exposure Followed by Ambient Curing

**Table 2:** Dataset ranges for training and testing the ANN models.

S.No	Mix-Design Influencing Factors	Unit	Training			Testing		
			Min.	Max.	Std. Dev.	Min.	Max.	Std. Dev.
1.	Compressive Strength	MPa	13.37	87.3	18.23	17.21	76.33	19.68
2.	Tensile Strength	MPa	1.55	6	1.21	1.6	5.3	1.27
3.	FA Content	kg/m^3^	0	1620	306.75	425	1246.1	291.42
4.	GGBS Content	kg/m^3^	0	562.8	155.58	0	320	147.77
5.	Sand Content	kg/m^3^	0	1172	396.56	0	1172	374.54
6.	Activator/Binder ratio	--	0.364	1.3	0.28	0.3988	1.3	0.27
7.	PVA Fiber	V_f_(%)	0.5	3	0.66	0.5	3	0.79
8.	Curing Temperature x Hours	°C.h	22	1440	496.13	25	1440	564.79
9.	Ambient Curing Duration	days	3	70	19.69	3	28	10.91

**Table 3:** Correlation coefficient of best ANN-I models during training.

ANN Model	R			
	Training	Self-Validation	Self-Testing	Overall
ANN [2:16:16:7]	0.91	0.91	0.87	0.90
ANN [2:16:25:7]	0.80	0.75	0.85	0.79
ANN [2:16:16:8:7]	0.81	0.95	0.88	0.83
ANN [2:16:16:25:7]	0.82	0.92	0.93	0.86
ANN [2:16:32:16:7]	0.86	0.91	0.87	0.86

**Table 4:** Regression analysis results of mix design prediction (**a**) Prediction of fly ash content (kg/m^3^); (**b**) prediction of GGBS content (kg/m^3^); (**c**) prediction of sand content (kg/m^3^); (**d**) prediction of activator–binder ratio; (**e**) prediction of PVA fiber V_f_ (%); (**f**) prediction of curing temperature x hrs (°C.h); (**g**) prediction of ambient curing (days).

(**a**)												
**Ref**		[39]	[40]	[40]	[44]	[46]	[48]	[49]	[35]	[47]	[48]	**R^2^**
	**Exp**	938	1134	1246.1	653.84	607.14	510	520	525	510	425	
**ANN**												
16-16		994.3	1356	1327.8	1029.7	331.7	494.2	537.8	817.1	196.1	393.6	0.74
16-25		1126.7	820.3	1445	1030.6	509.2	450	1574.5	632.2	478.9	169.3	0.28
16-16-25		931.2	908	990.1	882.7	776.2	414.7	1063.2	966.2	421.5	489.8	0.27
16-16-8		907.4	1278.4	881	1040.9	674.6	1196.4	894.7	428.2	694.7	500.2	0.23
16-32-16		815.3	1163.1	879.3	824.2	752.1	241.5	835.7	757.5	877.3	565.7	0.35
(**b**)												
**Ref**		[39]	[40]	[40]	[44]	[46]	[48]	[49]	[35]	[47]	[48]	**R^2^**
	**Exp**	235	252	0	0	0	320	0	0	320	0	
**ANN**												
16-16		228.2	560.8	0	0	0	324.6	0	0	29.6	0	0.48
16-25		562.4	356.8	15.9	0	0	470.6	519.2	0	513.9	0	0.58
16-16-25		122.7	124	87.6	62.5	46.5	294	89.7	76.8	378.8	158.7	0.62
16-16-8		64.8	66	44	34.6	172.3	150.7	63.9	106.8	246.5	80.2	0.23
16-32-16		150.2	84.2	47.1	119.3	63.1	314.5	40.2	103	426	213.3	0.49
(**c**)												
**Ref**		[39]	[40]	[40]	[44]	[46]	[48]	[49]	[35]	[47]	[48]	**R^2^**
	**Exp**	1172	0	392.3	281.53	348.57	0	275.9	278.6	0	800	
**ANN**												
16-16		1172	0	444.2	308.9	0.1	0	0	10.2	47.4	1001.4	0.85
16-25		0	0	1113.2	308.9	0.8	0	1085.2	193.9	1065.1	0	0.07
16-16-25		955.2	10.6	723.8	33.7	339.4	28.9	302.2	732.8	33.3	819	0.7
16-16-8		1164.2	117.7	869.9	212.2	192.4	804.9	675.6	83	118.5	902.4	0.46
16-32-16		1073	0.3	527	0.9	1.1	0.2	84.6	1020.6	690.1	560.6	0.31
(**d**)												
**Ref**		[39]	[40]	[40]	[44]	[46]	[48]	[49]	[35]	[47]	[48]	**R^2^**
	**Exp**	1.3	0.3988	0.471	0.45	0.4	0.42	0.53	0.55	0.42	0.6	
**ANN**												
16-16		0.8	0.41	0.46	0.39	0.46	0.5	0.55	0.46	0.73	0.69	0.44
16-25		0.39	0.39	0.46	0.36	0.37	0.37	0.36	0.67	0.47	1.2	0
16-16-25		1.09 *	1.09 *	1.04 *	1.01 *	1.08 *	1.18 *	1.11 *	1.03 *	1.16 *	1.21 *	0
16-16-8		1.24 *	0.5	0.88	0.38	0.46	0.72	0.43	1.18 *	0.48	1.18 *	0.41
16-32-16		1.09 *	1.24 *	1.24 *	1.26 *	1.26 *	1.05 *	1.29 *	1.18 *	1.24 *	1.05 *	0.21
(**e**)												
**Ref**		[39]	[40]	[40]	[44]	[46]	[48]	[49]	[35]	[47]	[48]	**R^2^**
	**Exp**	1.5	2	2	2	1.5	0.5	1.5	1.1	1	3	
**ANN**												
16-16		1.2	1.8	1.5	2.3 *	2.2 *	1.3	1.3	1.3	1.7	1.8	0.21
16-25		3.0 *	1.3	0.1	1.9	0.4	0.5	0	1.1	3.0 *	1	0.01
16-16-25		0.7	2.6 *	0.6	2.6 *	2.5 *	2.4 *	1.1	0.6	2.1 *	1	0.06
16-16-8		0.1	1.6	0.4	1	1.6	0.5	0.7	0.2	1.1	0.1	0
16-32-16		1.4	2.7 *	1.5	2.5 *	2.7 *	2.2 *	1.8	2.3 *	1.5	2	0.01
(**f**)												
**Ref**		[39]	[40]	[40]	[44]	[46]	[48]	[49]	[35]	[47]	[48]	**R^2^**
	**Exp**	25	25	240	1440	1440	25	160	160	25	320	
**ANN**												
16-16		23	22	23	1440	546	22	1068	69	23	479	0.46
16-25		22	28	22	1440	25	22	22	22	22	22	0.43
16-16-25		41	525	63	1436	1415	37	367	77	23	39	0.86
16-16-8		100	158	251	1436	33	34	943	119	24	160	0.23
16-32-16		26	1179	86	1062	1398	40	645	61	22	42	0.47
(**g**)												
**Ref**		[39]	[40]	[40]	[44]	[46]	[48]	[49]	[35]	[47]	[48]	**R^2^**
	**Exp**	7	28	28	3	28	7	7	7	28	14	
**ANN**												
16-16		59	70	68	3	60	4	3	4	7	3	0.37
16-25		70	20	70	3	70	47	65	56	70	70	0.06
16-16-25		30	9	33	7	9	5	22	32	34	13	0.02
16-16-8		14	9	10	5	7	14	6	30	12	18	0.06
16-32-16		41	5	36	5	5	7	26	34	42	19	0

* indicates the values that can be “adjusted”.

**Table 5:** ANN-II model testing with regression analysis.

Ref	Compressive Strength (MPa)			Tensile Strength (MPa)		
	Experimental Data	Predicted Data	RMSE	Experimental Data	Predicted Data	RMSE
[39]	29.1	29.4	0.33	2.64	2.90	0.26
[40]	59.6	57.5	2.09	4.2	5.09	0.11
[38]	20.9	20.7	0.16	3.2	2.68	0.52
[44]	56.8	61.7	4.86	5	4.07	0.93
[45]	43.1	28.3	14.82	5.3	4.46	0.84
[48]	53.5	48.8	4.72	1.6	1.52	0.08
[49]	22.06	15.7	6.36	3.7	1.75	1.95
[35]	17.21	14.1	3.07	2.48	1.85	0.63
[48]	76.33	72.7	3.59	4.4	3.79	0.61
[47]	37.2	36.3	0.90	1.97	1.84	0.13
**Mean**	**R^2^ = 0.936**	**RMSE = 4.09**		**R^2^ = 0.80**	**RMSE = 0.61**	

**Table 6:** ‘Tacked-together’ mix design.

Output/Mix Factor	ANN Model	‘Tacked-Together’ Mix Design										R^2^
Fly Ash	16-16	994.3	1356.0	1327.8	1029.7	331.7	494.2	537.8	817.1	196.1	393.6	0.74
GGBS	16-16-25	122.7	124.0	87.6	62.5	46.5	294.0	89.7	76.8	378.8	158.7	0.62
Sand	16-16	1172.0	0.0	444.2	308.9	0.1	0.0	0.0	10.2	47.4	1001.4	0.85
Act/Bin	16-16	0.80	0.41	0.46	0.39	0.46	0.50	0.55	0.46	0.73	0.69	0.44
PVA *	16-16	1.2	1.8	1.5	2 *	2 *	1.3	1.3	1.3	1.7	1.8	0.21
CT * Hrs	16-16-25	41	525	63	1436	1415	37	367	77	23	39	0.86
Ambient	16-16	59	70	68	3	60	4	3	4	7	3	0.37

* Adjusted.

**Table 7:** (**a**) Cross-validation of ANN-I models upon the prediction of compressive strength; (**b**) cross-validation of ANN-I models upon the prediction of tensile strength.

(**a**)											
**Ref**	**Experimental CS**	**ANN 16-16**	**RMSE**	**ANN 16-25**	**RMSE**	**ANN 16-16-25**	**RMSE**	**ANN 16-16-8**	**RMSE**	**ANN 16-32-16**	**RMSE**
[39]	29.1	70.40	41.30	24.16	4.94	23.84	5.26	13.64	15.46	37.84	8.74
[40]	59.6	86.53	26.93	75.77	16.17	50.17	9.43	49.48	10.12	74.84	15.24
[38]	20.9	30.02	9.12	13.91	6.99	27.15	6.25	14.65	6.25	23.15	2.25
[44]	56.8	64.21	7.41	61.48	4.68	77.38	20.58	45.14	11.66	63.45	6.65
[45]	43.1	17.41	25.69	32.96	10.14	23.43	19.67	21.83	21.27	57.55	14.45
[48]	53.5	44.32	9.18	79.91	26.41	34.79	18.71	25.12	28.38	47.63	5.87
[49]	22.06	14.59	7.47	23.03	0.97	33.83	11.77	32.12	10.06	22.39	0.33
[35]	17.21	14.79	2.42	17.12	0.09	25.22	8.01	19.55	2.34	31.92	14.71
[48]	76.33	13.92	62.41	74.58	1.75	84.36	8.03	32.43	43.90	82.33	6.00
[47]	37.2	18.67	18.53	34.84	2.36	15.13	22.07	13.46	23.74	26.14	11.06
**Mean RMSE**			**21.05**		**7.45**		**12.98**		**17.32**		**8.53**
(**b**)											
**Ref**	**Experimental TS**	**ANN 16-16**	**RMSE**	**ANN 16-25**	**RMSE**	**ANN 16-16-25**	**RMSE**	**ANN 16-16-8**	**RMSE**	**ANN 16-32-16**	**RMSE**
[39]	2.64	3.94	1.30	2.78	0.14	2.12	0.52	2.27	0.37	2.59	0.05
[40]	4.2	5.74	1.54	5.11	0.91	3.51	0.69	2.33	1.87	5.10	0.90
[38]	3.2	4.40	1.20	3.04	0.16	2.01	1.19	1.76	1.44	2.00	1.20
[44]	5	4.38	0.62	4.42	0.58	5.33	0.33	4.64	0.36	4.91	0.09
[45]	5.3	3.01	2.29	5.30	0.00	4.42	0.88	2.23	3.07	5.24	0.06
[48]	1.6	3.20	1.60	1.56	0.04	3.53	1.93	1.96	0.36	3.35	1.75
[49]	3.7	3.21	0.49	3.69	0.01	2.52	1.18	3.06	0.64	3.22	0.48
[35]	2.48	1.86	0.62	2.92	0.44	1.96	0.52	2.70	0.22	2.40	0.08
[48]	4.4	1.88	2.52	4.30	0.10	5.27	0.87	2.85	1.55	5.40	1.00
[47]	1.97	1.78	0.19	2.28	0.31	1.90	0.07	2.04	0.07	2.84	0.87
**Mean RMSE**			**1.24**		**0.27**		**0.82**		**0.99**		**0.65**

**Table 8:** Cross-validation of tacked-together outputs upon the prediction of compressive and tensile strength.

Experimental CS	Predicted CS	RMSE	Experimental TS	Predicted TS	RMSE
29.1	40.39	11.29	2.64	2.69	0.05
59.6	57.72	1.88	4.2	4.59	0.39
20.9	17.81	3.09	3.2	3.10	0.10
56.8	55.31	1.49	5	3.54	1.46
43.1	44.59	1.49	5.3	5.72	0.42
53.5	57.71	4.21	1.6	3.74	2.14
22.06	28.91	6.85	3.7	2.97	0.73
17.21	26.01	8.80	2.48	2.60	0.12
76.33	83.12	6.79	4.4	4.81	0.41
37.2	25.23	11.97	1.97	1.89	0.08
**Mean**	**RMSE = 5.76**			**RMSE = 0.59**	

## Data Availability

The datasets generated during and/or analysed during the current study are available from the corresponding author on reasonable request.
